# Topsoil Bacterial Community Changes and Nutrient Dynamics Under Cereal Based Climate-Smart Agri-Food Systems

**DOI:** 10.3389/fmicb.2020.01812

**Published:** 2020-07-28

**Authors:** Madhu Choudhary, Hanuman S. Jat, Ashim Datta, Parbodh C. Sharma, Balaji Rajashekar, Mangi L. Jat

**Affiliations:** ^1^ICAR-Central Soil Salinity Research Institute (CSSRI), Karnal, India; ^2^International Maize and Wheat Improvement Center (CIMMYT), New Delhi, India; ^3^Celixa, Bengaluru, India

**Keywords:** bacterial diversity, relative abundance, DNA sequencing, available nutrients, cereal-based systems

## Abstract

Soil microorganisms play a critical role in soil biogeochemical processes, nutrient cycling, and resilience of agri-food systems and are immensely influenced by agronomic management practices. Understanding soil bacterial community and nutrient dynamics under contrasting management practices is of utmost importance for building climate-smart agri-food systems. Soil samples were collected at 0–15 cm soil depth from six management scenarios in long-term conservation agriculture (CA) and climate-smart agriculture (CSA) practices. These scenarios (Sc) involved; ScI-conventional tillage based rice-wheat rotation, ScII- partial CA based rice-wheat-mungbean, ScIII- partial CSA based rice-wheat-mungbean, ScIV is partial CSA based maize-wheat-mungbean, ScV and ScVI are CSA based scenarios, were similar to ScIII and ScIV respectively, layered with precision water & nutrient management. The sequencing of soil DNA results revealed that across the six scenarios, a total of forty bacterial phyla were observed, with *Proteobacteria* as dominant in all scenarios, followed by *Acidobacteria* and *Actinobacteria*. The relative abundance of *Proteobacteria* was 29% higher in rice-based CSA scenarios (ScIII and ScV) and 16% higher in maize-based CSA scenarios (ScIV and ScVI) compared to conventional-till practice (ScI). The relative abundance of *Acidobacteria* and *Actinobacteria* was respectively 29% and 91% higher in CT than CSA based rice and 27% and 110% higher than maize-based scenarios. Some taxa are present relatively in very low abundance or exclusively in some scenarios, but these might play important roles there. Three phyla are exclusively present in ScI and ScII i.e., *Spirochaetes*, *Thermi*, and *Euryarchaeota*. Shannon diversity index was 11% higher in CT compared to CSA scenarios. Maize based CSA scenarios recorded higher diversity indices than rice-based CSA scenarios. Similar to changes in soil bacterial community, the nutrient dynamics among the different scenarios also varied significantly. After nine years of continuous cropping, the soil organic carbon was improved by 111% and 31% in CSA and CA scenarios over the CT scenario. Similarly, the available nitrogen, phosphorus, and potassium were improved by, respectively, 38, 70, and 59% in CSA scenarios compared to the CT scenario. These results indicate that CSA based management has a positive influence on soil resilience in terms of relative abundances of bacterial groups, soil organic carbon & available plant nutrients and hence may play a critical role in the sustainability of the intensive cereal based agri-food systems.

## Introduction

Soil is the foundation of any agricultural production system; keeping the soil in a good health is crucial to produce desired food, feed, and fiber to meet the growing population requirements on a sustainable basis. Indo-Gangetic plains (IGP) of South Asia is not only a highly productive and intensively cultivated region but also the most populous region of the world and thereby posing a significant pressure on natural resources. Over the past few decades, the mounting pressure to produce more food using conventional resource management practices has led to the degradation of the natural resources (soil, water, environment). The degradation process will be more aggressive in the future and may have more impact under the growing challenges of climate change (Jat et al., 2019b). Since the intensive cereal systems of Indian western IGP will continue to serve as the basis for food security, the overexploitation of resources in that process has raised serious concerns for the sustainability of natural resources especially groundwater and soil health. Ensuring soil’s resilience is critical to provide long-term sustainability to food security in the region. In this respect, different soil and crop management practices such as conservation agriculture and precision water and nutrient management have a significant role to play not only for producing more from less but also to mediate the soil processes for improving soil biological, chemical and physical properties (Choudhary et al., 2018b; Jat et al., 2018).

In Indian western IGP, conventional management practices of growing rice-wheat rotation are labor, water, and energy-intensive and accelerate the loss of soil organic carbon (SOC) and soil quality (chemical, physical and biological) deterioration (Jat et al., 2018). Increasing costs of production and changes in the subsidy policies of the Government are forcing the farmers to use chemical fertilizers in favor of N at the cost of P, K and other micro-nutrients. These are the causes of soil nutrient imbalances, loss of soil fertility, decreasing nutrient use efficiency and increasing cost of nutrient management. Conventional agriculture practices like repeated tillage, open-field burning of crop residues and over-pumping of groundwater in the monotonous rice-wheat cropping systems are the major drivers for the unsustainability of the natural resources especially soil and environmental quality, and groundwater table (Lohan et al., 2018; Jat et al., 2019b). Moreover, the productivity, growth, and sustainability of agricultural crops depend upon soil health status defined by a set of measurable physical, chemical, and biological attributes as well as functional soil processes controlled by management and climate change drivers. The climate change induced extreme weather events are increasing and projected to be growing at multiple rates in the future if effective measures are not taken. Therefore, adapting agriculture and crop production systems to these extreme climate events are required to build sustainable agri-food systems for the future (Sapkota et al., 2019).

There are a wide range of management practices having the potential to increase resource use efficiency, improve adaptive capacity while reducing the environmental footprint from the production system, and are defined as climate-smart agriculture (CSA) practices (FAO, 2010; Jat et al., 2016). Among different CSA practices; zero-till direct drilling, crop residue retention, crop diversification, and precision nutrient and water management are considered key to positively influence crop productivity and soil health (Ghimire et al., 2017; Jat et al., 2018, 2019a,b; Datta et al., 2019) while adapting to climatic risks and reducing green house gas (GHG) emissions (Sapkota et al., 2015; Jat et al., 2016). However, the application of CSA practices in isolation may or may not play its potential role in adapting to climate risks in the intensive agri-food systems of the IGP. Moreover, recycling of crop residues with viable *in situ* management practices in largely mechanized harvesting of intensive rice-wheat rotation is a must for soil’s resilience and system sustainability (Lohan et al., 2018; Shyamsundar et al., 2019). Therefore, layering of these CSA practices in optimal combinations may help in adapting to climate risks and building resilience to climate variability and ensure food security (Aryal et al., 2016; Kakraliya et al., 2018; Jat et al., 2019b).

For a sustainable agri-food system, soil resilience is one of the key foundations. Soil microorganisms are inevitable constituents of the soil ecosystem and play critical roles in its essential processes and critical functions, mainly nutrient cycling, soil organic matter dynamics etc. (Nannipieri et al., 2003; Kibblewhite et al., 2007) and making soil resilient toward climate change. It is therefore important to study the soil microbial community composition and to improve knowledge on its role in the agro-ecosystem. Under Intensive cereal-based systems of South Asia, several studies have been conducted to understand the effects of CSA practices on soil carbon pools (Jat et al., 2019a), soil quality (Jat et al., 2020), productivity and profitability (Kakraliya et al., 2018; Jat et al., 2019b) and their role in adaptation to climatic risks (Kakraliya et al., 2018) as well as GHG mitigation (Sapkota et al., 2019) but the studies on soil microbial structure and community changes and their role in building the resilience are limited under CSA practices. Effect of management practices on soil microbial populations has been studied for a long time but most of these studies were done by the methods of culturing, substrate utilization, and phospholipid fatty acid analysis (Doran, 1980; Parkinson and Coleman, 1991; Govaerts et al., 2008; Jangid et al., 2008). Due to limitations of these methods only a small portion of microbial communities can be studied (Atlas and Bartha, 1998; Handelsman, 2004; Douterelo et al., 2014). With every day advancing in sequencing technologies and with the availability of high throughput sequencing platforms, studies can be done more intensively on microbial communities. These sequencing approaches can offer a wider understanding of the complex structures of microbial communities at the different levels of microbial taxa (Cardenas and Tiedje, 2008).

Globally a number of studies has been undertaken on the effects of different agricultural management practices on the taxonomic diversity of bacterial communities by throughput sequencing (Lienhard et al., 2014; Degrune et al., 2016; Wang et al., 2016; Piazza et al., 2019; Sun et al., 2020). They reported that soil and crop management practices significantly influenced soil microbial community structure and abundance. Management practices not only manipulate soil biological properties but also chemical properties as both are interlinked to each other. The microorganisms play a critical role in regulating soil carbon dynamics by adopting different mechanisms (Zhou et al., 2012). With the warming conditions due to climate change scenarios, Li et al. (2019) projected a decrease in carbon use efficiency and an increase in microbial biomass turnover rate. Whereas, for stability and resilience, the interaction between communities also changes, which can influence the gene expression of single species (Jansson and Hofmockel, 2020). With the changing soil conditions either by climate or by different management, soil microorganisms respond by acclimation and adaption. But most of these studies were undertaken in isolation to understand and quantify the effect of one or two factors/treatments/practices like tillage and crop residue on microbial communities. However, under the growing complexity of climate change effects on agriculture, the application of portfolio of multiple practices (includes a wide range of practices like crop rotation, tillage, crop establishment, crop residue retention, precision nutrient and water management), called CSA, are needed. Under such portfolio of CSA practices, understanding the role of soil bacterial community and nutrient dynamics in building resilience would further help to advance the science to address the climate change issues in agri-food systems.

For the comprehensive understanding of microbial community composition, under different CSA practices involving crop rotation, tillage, crop establishment, residue, nutrient, and water management, we assessed their effects on the bacterial community structure, diversity and nutrients availability in a long-term experiment under semi-arid climates of Indian western IGP. The study was carried out with specific objectives (1) to study the shift in soil bacterial diversity and community composition in rice/maize-based agri-food systems under CSA practices; (2) quantify the availability of soil macro and micronutrients changes under CSA practices over farmers’ management, and; (3) establish the relationships between the composition of the bacterial community and nutrients under CSA in cereal-based food systems.

## Materials and Methods

### Study Site and Experimental Design

A long-term production scale fixed plot experiment was started in 2009 at the Indian Council of Agricultural Research -Central Soil Salinity Research Institute (29.42°N latitude, 76.57°E longitude, and at an elevation of 243 m above msl), Karnal, Haryana, India. The experiment initially consisted of four management scenarios (Jat et al., 2020) but later in 2016 two more scenarios (ScV and ScVI) were introduced by splitting ScIII and ScIV into two equal parts (Jat et al., 2019b). All the scenarios were varied with residue, tillage, crop, irrigation, and nutrient applications. Six scenarios (Sc) included were: (i) farmers’ practice (ScI, conventional-till (CT) rice-CT wheat); (ii) partial conservation agriculture/CA (ScII, CT rice-Zero tillage (ZT) wheat-ZT mungbean with flood irrigation); (iii) rice-based partial climate-smart agriculture (CSA) (ScIII, ZT rice-ZT wheat-ZT mungbean with flood irrigation); (iv) maize-based partial CSA (ScIV, ZT maize-ZT wheat-ZT mungbean with flood irrigation); (v) rice-based full CSA (ScV, ZT rice-ZT wheat-ZT mungbean with SDI); and (vi) maize-based full CSA (ScVI, ZT maize-ZT wheat-ZT mungbean with SDI). ScIII and ScIV were based on principals of CA practices and called partial CSA. However, in ScV and ScVI, in addition to the ScIII and ScIV, irrigation water and N were also precisely managed using subsurface drip irrigation (SDI) and designated full CSA. Scenarios were structured in a randomized complete block design and replicated thrice. The description of scenarios is listed in Table 1.

**TABLE 1:** Drivers of agricultural change, crop rotation, tillage, crop establishment method, and residue management of different scenarios.

**Scenario**	**Scenario depiction**	**Drivers of change**	**Crop rotations**	**Tillage**	**Crop establishment method**	**Residue management**	**Nutrient management (NPK, kg/ha)**	**Water management**
ScI	CT-RW	Business as usual (Farmer’s Practice)	Rice-Wheat- Fallow	CT-CT	Rice: Transplantin Wheat: Broadcast	All residue removed	Rice: 175 + 58 + 0 Wheat: 150 + 58 + 0	Rice: Continuous flooding of 5-cm depth for 1 month, followed by irrigation applied at hair-line crack Wheat: Need based irrigation or at critical crop growth stages
ScII	Partial CA-RW	Increase food production, income & nutrition through intensification and best management practices	Rice-Wheat-Mungbean	CT-ZT-ZT	Rice: Transplanting Wheat: Drill seeding Mungbean: Drill/relay	Full (100%) rice and anchored wheat residue retained on soil surface; full mungbean residue incorporated	Rice: 151 + 58 + 60 Wheat: 151 + 64 + 32 Mungbean: o + o + o	Rice: Continuous flooding of 5-cm depth for first 15-20 days after transplanting ‘fb’ irrigation at −40 to −50 kPa matric potential at 15-cm depth till 1 wk before flowering
								‘Wheat: Flood irrigation at −40 to −50 kPa matric potential
ScIII	Partial CSA-RW	Deal with rising scarcity of labor, water, energy, malnutrition, degrading soil health and emerging climatic variability	Rice-Wheat-Mungbean Wheat: Drill seeding	ZT-ZT-ZT	Rice: Drill seeding Mungbean: Drill/relay	Full (100%) rice and Mungbean; anchored wheat residue retained on soil surface	Rice: 162 + 64 + 62 Wheat: 151 + 64 + 32 Mungbean: o + o + o	Rice: Kept soil wet for first 20 days ‘fb’ irrigation at −20 to −30 kPa matric potential Wheat: Flood irrigation at −40 to −50 kPa matric potential
ScIV	Partial CSA-MW	Sustainable intensification (SI) with futuristic cropping system to deal with same issues as in scenario 3	Maize-wheat- Mungbean	ZT-ZT-ZT	Maize: Drill seeding Wheat: Drill seeding Mungbean: Drill/relay	Maize (65%) and full mungbean; anchored wheat residue retained on soil surface	Maize: 174 + 64 + 62 Wheat: 151 + 64 + 32 Mungbean: o + o + o	Flood Irrigation at −50 kPa in maize and −40 to −50 kPa matric potential in wheat
ScV	CSA-RW	SI of RW system with CA + to deal with same issues as in scenario 3	Rice-Wheat-Mungbean	ZT-ZT-ZT	Same as in scenario 3	Same as in scenario 3	Rice: 130 + 64 + 62 Wheat: 121 + 64 + 32 Mungbean: o + o + o N in rice- 8 splits & wheat- 4 splits through SSD Fertigation	Sub surface drip irrigation (SSDI) at −20 to −30 kPa in rice and −40 to −50 kPa matric potential in wheat
ScVI	CSA-MW	SI of MW systems through CA + to deal same issues as in scenario 3	Maize-wheat- Mungbean	ZT-ZT-ZT	Same as in scenario 4	Same as in scenario 4	Maize: 139 + 64 + 62 Wheat: 121 + 64 + 32 Mungbean: o + o + o N in maize- 3 splits & wheat- 4 splits through SSD Fertigation	Sub surface drip irrigation (SSDI) at −50 kPa in maize and −40 to −50 kPa matric potential in wheat

**Where*: CT, conventional tillage; ZT, zero till; CA, conservation agriculture; SI, sustainable intensification; SSD, sub surface drip; SSDI, sub surface drip irrigation; N, nitrogen; P, phosphorus; K, potassium.*

### Soil Sampling and Analysis

Soil samples (0–15 cm) were collected from six scenarios replicated thrice (a total of 18 samples) after harvesting of the wheat crop in May 2018. Crop residues were removed from the soil surface and samples were taken randomly aseptically using an auger. After sampling, soil samples were sieved by 2-mm sieve and were divided into two parts. One part was immediately transferred to the laboratory for DNA extraction and the other part was air-dried, ground, and stored in plastic containers for chemical analysis.

Soil electrical conductivity (EC) and pH in the soil to water ratio of 1:2 were determined by standard methods (Jackson, 1973). The total carbon content of the soils was determined using the CHNS Vario El III analyzer (Elementar, Germany). As inorganic carbon was negligible, the total carbon represents total organic carbon and designated as soil organic carbon (SOC). The available N in soil was determined by the method of Subbiah and Asija (1956). Available Phosphorus (Olsen P) was determined by the method outlined by Olsen et al. (1954). Available Potassium (K) in soil was determined by flame photometer using neutral 1*N* ammonium acetate extractant as described by Jackson (1973). Available Sulfur (S) was estimated by the turbidimetric barium chloride method given by Chesnin and Yien (1951). Available (DTPA-extractable) Iron (Fe), Manganese (Mn), Zinc (Zn) and Copper (Cu) in the soil samples were estimated by extracting the soils with 0.005M DTPA + 0.01M CaCl_2_ + 0.1M TEA solution adjusted to pH 7.3 (soil: extractant ratio 1:2) and micronutrient cations in the extract were determined using atomic absorption spectrophotometer (Lindsay and Norvell, 1978).

### Extraction and Sequencing of Soil DNA

From the soil samples, DNA was extracted by MO BIO’s PowerSoil DNA Isolation Kit as per the instructions of the manufacturer. The DNA quantity and quality was measured by Nanodrop spectrophotometer (ThermoFisher Scientific, United States). Amplicon sequencing libraries for V3-V4 fragments of the 16S rRNA gene were prepared. The protocol also includes overhang adapter sequences that were appended to the primer pair sequences for compatibility with Illumina index and sequencing adapters. About 30 ng of template DNA was amplified for 26 cycles using KAPA HiFi Hot Start PCR Kit (KAPA Biosystems Inc., Boston, MA, United States). The forward and reverse primer concentrations were kept at 0.2 μM each. The amplicons were confirmed on 1.5% agarose gel electrophoresis. The second round of PCR was performed for 10 cycles to add appropriate sample-specific indexes and Illumina flow cell-specific sequences. We have used Illumina Nextera XT v2 Index Kit (Illumina, United States) and the PCR was carried out with HiFi Hot Start PCR Kit.

The second-round PCR products (sequencing libraries) were purified using Ampure XP magnetic beads (Beckman Coulter, United States) and concentrations were measured using Qubit dsDNA HS assay (Thermo Fisher Scientific, United States). The sample libraries were normalized based on the Qubit concentrations and proceeded for sequencing in Illumina MiSeq 300 paired-end chemistry. The forward primer was constructed with the Illumina i5 adapter (5′ – AATGATACGGCGACCACCGAGATCTACAC[i5]TCGTCGGC AGCGTC), and the reverse fusion primer was constructed with the Illumina i7 adapter (5′ – CAAGCAGAAGACGG CATACGAGAT[i7]GTCTCGTGGGCTCGG).

V3V4_Forward 341FCCTACGGGNGGCWGCAG

V3V4_Reverse 805RGACTACHVGGGTATCTAATCC

The libraries were normalized and pooled for multiplex sequencing. Finally, these pools were quantified using Qubit dsDNA HS assay and fluorometer (Thermo Fisher Scientific, MA, United States) and then diluted to 2 nM final concentration using Resuspension Buffer (RSB -Illumina, CA, United States). The normalized sample was denatured for 5 minutes using 0.2 N NaOH and neutralized by HT1 Buffer (Illumina, CA, United States). Denatured libraries were further diluted to 13 pM concentration for loading. Samples were then loaded into an Illumina MiSeq v3 600 cycles cartridge (Illumina, CA, United States). The flow cell and the PR2 buffer were placed in the designated slots in the machine and the run was performed in paired-end mode with 275 bp read length for each of forward (Read 1) and reverse (Read 2) reads.

### Bioinformatics and Statistical Analysis of Data

After completion of the sequencing run, the data were demultiplexed using bcl2fastq software v2.20 and Fast Q files were generated based on the unique dual barcode sequences. The sequencing quality was assessed using Fast QC v0.11.8 software. The paired-end reads were quality checked with FastQC (Available from S. Andrews at^1^) and the raw reads having primer sequence and high-quality bases (≥Q 30) were selected. The reads were further stitched (Aronesty, 2013) and analyzed by the QIIME 1 pipeline (Caporaso et al., 2010). The query sequences were clustered using the UCLUST method (Edgar, 2010) against a curated chimera free 16S rRNA database (DeSantis et al., 2006). RDP classifier (Wang et al., 2007) was used to assign the taxonomies to the clusters at ≥97% sequence similarity against the reference database which resulted in the generation of a biom file. Further sample wise and comparative analysis was performed. Alpha diversity- Shannon, Simpson, Chao, OTUs (Operational Taxonomic Units), and rarefaction curves were calculated based on the rarefied biom. Beta diversity was determined by principal coordinate analysis (PCoA) using both unweighted and weighted UniFrac metrics. The data were further subjected to analysis of variance (ANOVA) and analyzed using the general linear model (GLM) procedures of the SPSS Windows version 17.0 (SPSS Inc., Chicago, IL, United States). Treatment means were separated by Duncan Multiple Range Test at 5% level of significance.

The principal component analysis was done with the dataset (of 19 attributes) following Andrews et al. (2002) and Choudhary et al. (2018a). The principal components receiving high eigenvalues and variables with high factor loading were assumed to be variables that best represented system attributes. Therefore, only the PCs with eigenvalues > 0.9 and those that explained at least 5% of the variation in the data were examined. Only highly weighted variables within each PC were retained for the minimum data set (MDS). When more than one variable was retained under a single PC, multivariate Pearson’s correlation coefficients were employed to determine if the variables could be considered redundant and variables with the highest correlation sum were selected for the MDS (Andrews et al., 2002).

## Results

### Effect of Management Scenarios on α and β-Diversity

**FIGURE 1:**
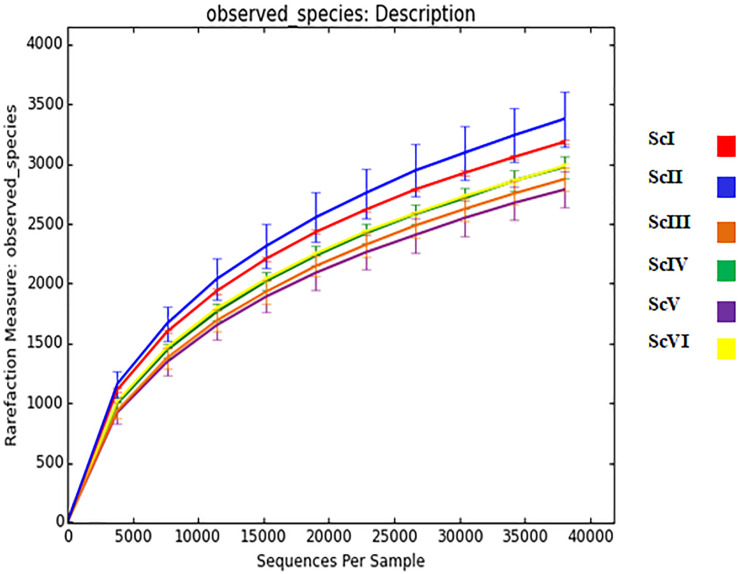
Rarefaction analysis of six soil samples having different agriculture management systems. *Where*; ScI, conventional rice-wheat system; ScII, partial CA based rice-wheat-mungbean system; ScIII, partial CSA based rice-wheat-mungbean system; ScIV, partial CSA based maize-wheat- mungbean system; ScV, full CSA based rice-wheat-mungbean system with sub surface drip irrigation; ScVI, full CSA based maize-wheat-mungbean system with sub surface drip irrigation.

**FIGURE 2:**
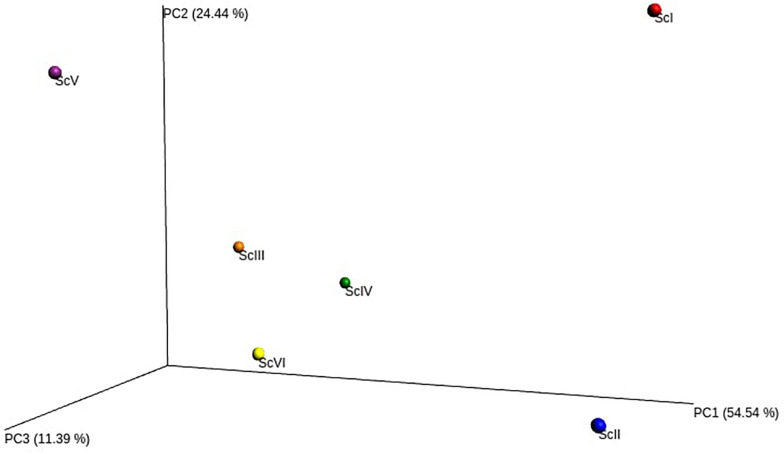
Principal coordinate analysis (PCoA) of soil bacterial β-diversity based on weighted UniFrac.

From the extracted DNA of 18 soil samples (6 scenarios each replicated thrice) a total of 5,601,598 raw reads were obtained for the V3-V4 region. After quality filtering, a total of 4,743,950 processed reads were obtained; from these 869,033 reads were utilized for bacterial identification. Sequences from all six scenarios have been submitted to NCBI with the Bio project: PRJNA563825. These reads were grouped according to their sequences and a total of 59,372 OTU were identified in six samples. Rarefaction analysis was done for observed species/OTUs with a minimum of 38000 sequences per sample (Figure 1). A significantly (p ≤ 0.05) higher number of OTU (3765 ± 127) and Chao1 (5306 ± 108) were recorded in ScII (Table 2). Shannon diversity index was recorded 11% higher in conventional tillage (CT) based scenarios (ScI and ScII) compared to those of CSA based scenarios (ScIII to ScVI). All diversity indices Shannon, Simpson, Chao1, and OTU have recorded higher in maize-based scenarios (ScIV and ScVI) compared to rice-based CSA scenarios (ScIII and ScV) (Table 2). The β**-**diversity matrix focuses on the difference in taxonomic abundance profiles from different samples. Beta diversity by weighted Unifrac method considers both sequences and abundance information and generates a distance matrix containing a dissimilarity value for each pairwise comparison for each sample (Figure 2). ScIII was observed to have less difference in taxonomy abundance with ScIV, ScVI, and ScV. In contrast, ScV had a higher difference in taxonomic abundance with ScII and ScI. On average ScI, ScII, ScV had higher difference while ScIII, ScIV, ScVI had less difference.

**TABLE 2:** Bacterial diversity indices of six scenarios for clusters at ≥97% sequence similarity against the reference database.

**Scenario^a^**	**Shannon**	**Simpson**	**Chao1**	**OTU**
ScI	9.20 ± 0.09a^b^	0.993 ± 0.00a	4643 ± 97b	3278 ± 43b
ScII	9.37 ± 0.35a	0.993 ± 0.00a	5306 ± 108a	3765 ± 127a
ScIII	8.24 ± 0.33b	0.978 ± 0.01ab	4551 ± 36b	3055 ± 48b
ScIV	8.58 ± 0.19ab	0.984 ± 0.00a	4688 ± 50b	3185 ± 62b
ScV	8.00 ± 0.40b	0.964 ± 0.01b	4446 ± 22b	3034 ± 60b
ScVI	8.76 ± 0.06ab	0.987 ± 0.00a	4670 ± 93b	3288 ± 69b

**Where*; ScI, conventional rice-wheat system; ScII, partial CA based rice-wheat-mungbean system; ScIII, partial CSA based rice-wheat-mungbean system; ScIV, partial CSA based maize-wheat-mungbean system; ScV, full CSA based rice-wheat-mungbean system with sub surface drip irrigation; ScVI, full CSA based maize-wheat-mungbean system with sub surface drip irrigation. ^*a*^Refer Table 1 for scenario description. ^*b*^Means followed by the same letters within each column are not statistically different (*p* ≤ 0.05, Duncan’s multiple range test). Values are the average of three replicates (*n* = 3); means ± standard error SE.*

### Bacterial Community Structure in Different Management Scenarios

**FIGURE 3:**
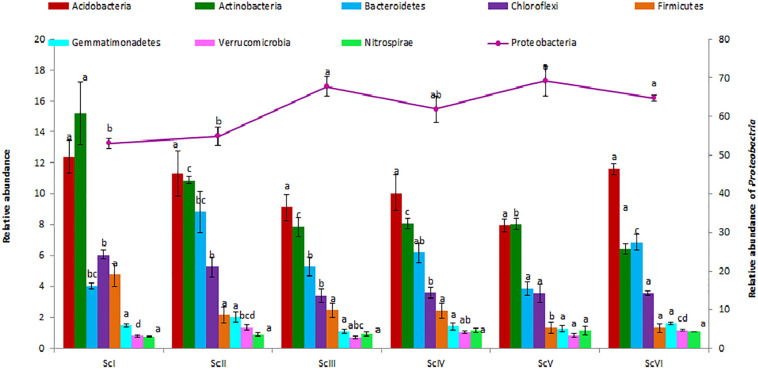
Distribution of different dominating phyla (> 1%) in different agriculture management scenarios. *Where*; ScI, conventional rice-wheat system; ScII, partial CA based rice-wheat-mungbean system; ScIII, partial CSA based rice-wheat-mungbean system; ScIV, partial CSA based maize-wheat-mungbean system; ScV, full CSA based rice-wheat-mungbean system with sub surface drip irrigation; ScVI, full CSA based maize-wheat-mungbean system with sub surface drip irrigation. The same letters within each column are not statistically different between scenarios for each phylum (*P* ≤ 0.05, Duncan’s multiple range test) and values are the average of three replicates (*n* = 3); means ± standard error SE.

A total of 40 phyla were observed in six scenarios of crop management. *Proteobacteria* was the dominant phylum in all scenarios followed by *Acidobacteria* and *Actinobacteria* (Figure 3). *Proteobacteria* ranged from 53.1 ± 1.23 (ScI) to 69.2 ± 3.91% (ScV). The relative abundance of *Proteobacteria* was 29% higher in rice-based CSA scenarios (ScIII and ScV) and 16% higher in maize-based CSA scenarios (ScIV and ScVI) over farmers’ practice (ScI). The relative abundance of *Acidobacteria* (ranged from 7.98 to 12.4%), and was not significantly different between scenarios (*p* ≤ 0.05). *Actinobacteria* was followed by *Acidobacteria* in ScII, ScIII, ScIV, and ScVI but in ScI and ScV *Actinobacteria* (15.2 ± 2.06 and 8.03 ± 0.38%) was higher than *Acidobacteria* (12.37 ± 1.06 and 7.98 ± 0.39%). *Actinobacteria* was 100% higher in farmers’ practice (ScI) and 43% in partial CA scenario (ScII) than CSA based scenarios (ScIII-VI). The relative abundance of *Acidobacteria* and *Actinobacteria* was respectively 29% and 91%, 27% and 110%, and 10% and 40% higher in farmers’ practice than rice-based CSA scenarios, maize-based scenarios, and partial CA scenario. The top five most abundant phyla, *Proteobacteria*, *Acidobacteria*, *Actinobacteria*, *Bacteroidetes*, and *Cholroflexi*, were represented by nearly 90% of the total sequences. The relative abundance of *Chloroflexi* was found significantly (*p* ≤ 0.05) higher in ScI (6.05 ± 0.28%) and ScII (5.27 ± 0.63%) than CSA based scenarios. The relative abundance of members of *Firmicutes* was significantly (*p* ≤ 0.05) higher in ScI (4.76 ± 0.75%) over other scenarios.

**FIGURE 4:**
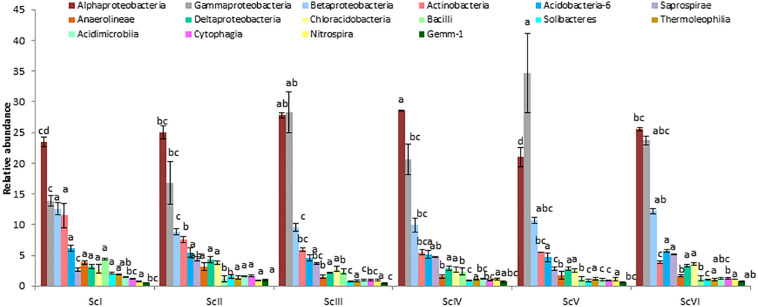
Relative abundance of classes (> 1%) in different agriculture management scenarios. *Where*; ScI, conventional rice-wheat system; ScII, partial CA based rice-wheat-mungbean system; ScIII, partial CSA based rice-wheat-mungbean system; ScIV, partial CSA based maize-wheat-mungbean system; ScV, full CSA based rice-wheat-mungbean system with sub surface drip irrigation; ScVI, full CSA based maize-wheat-mungbean system with sub surface drip irrigation. The same letters within each column are not statistically different (*P* ≤ 0.05, Duncan’s multiple range test) and values are the average of three replicates (*n* = 3); means ± standard error SE.

**FIGURE 5:**
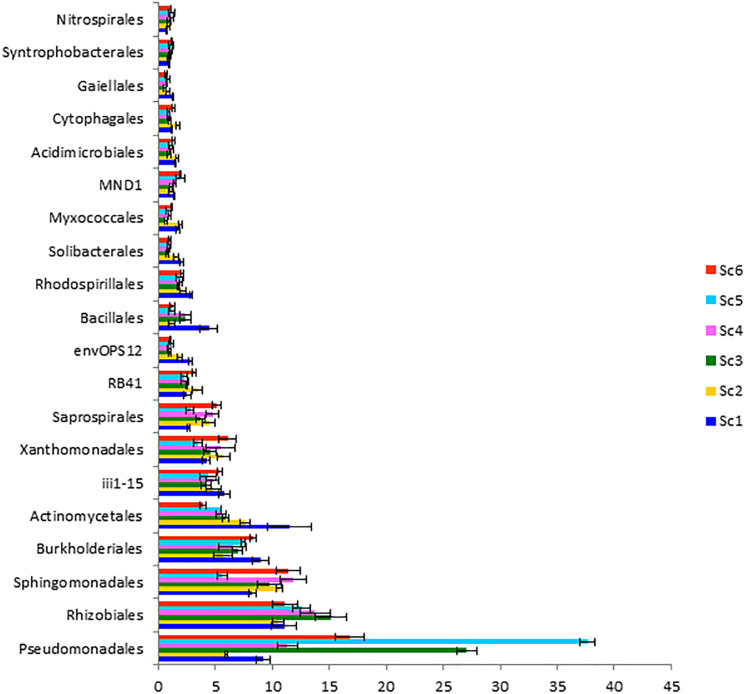
Distribution of dominating orders (> 1%) in different agriculture management scenarios. *Where*; ScI, conventional rice-wheat system; ScII, partial CA based rice-wheat-mungbean system; ScIII, partial CSA based rice-wheat-mungbean system; ScIV, partial CSA based maize-wheat-mungbean system; ScV, full CSA based rice-wheat-mungbean system with sub surface drip irrigation; ScVI, full CSA based maize-wheat-mungbean system with sub surface drip irrigation. The same letters within each column are not statistically different (*P* ≤ 0.05, Duncan’s multiple range test) and values are the average of three replicates (*n* = 3); means ± standard error SE.

Different relative abundances were also observed at the class level (Figure 4). *Alphaproteobacteria* was recorded highest in ScIV (28.55 ± 0.12%) followed by ScIII (27.78 ± 0.40%). However, the abundance of *Gammaproteobacteria* was higher in CSA based scenarios (20.57% – 41.16%) than partial CA (13.30%) and farmers’ practice (13.86%). These two classes *Alphaproteobacteria* and *Gammaproteobacteria* represented 37 to 62% sequences in the different scenarios. At the order level, relative abundance of *Pseudomonadales* was reported highest followed by *Rhizobiales* and *Sphingomonadales* (Figure 5). Among the scenarios, *Pseudomonadales* was significantly higher in rice-based CSA scenarios (ScIII and ScV) over other scenarios. It was 130% higher in rice-based over maize-based CSA scenarios (ScIV and ScVI), 251% higher in rice-based and 53% higher in maize-based CSA scenarios over farmers’ practice. *Rhizobiales* showed no significant differences (*p* ≥ 0.05) between scenarios. The relative abundance of *Sphingomonadales* was observed higher (51%) in maize-based CSA scenarios over rice-based CSA scenario. *Actinomycetales* were 121% higher in farmers’ practice over CSA-based scenarios (ScIII, ScIV, ScV, and ScVI) and 51% higher over partial CA-based scenario (ScII). In all six scenarios *Pseudomonadales*, *Rhizobiales*, *Sphingomonadales*, *Burkholderiales*, and *Actinomycetales* were the dominant orders, constituting approximately 49 -69% in different scenarios.

**FIGURE 6:**
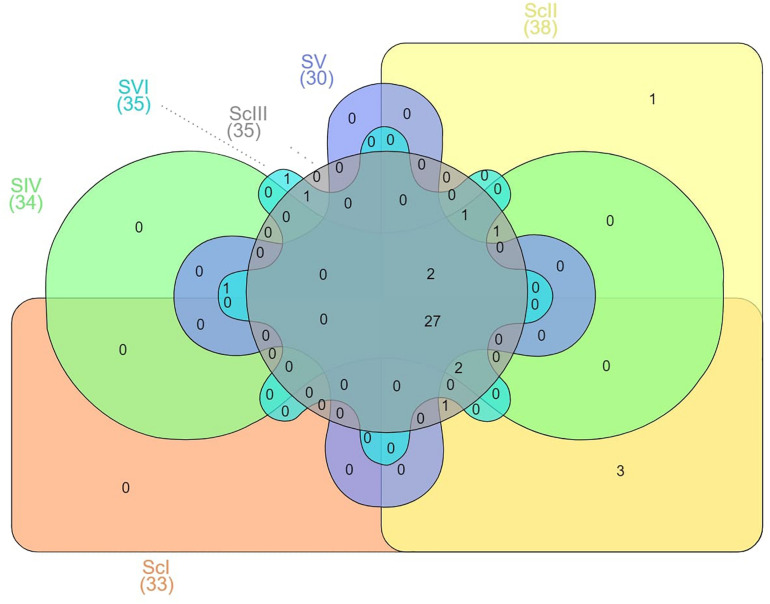
Venn graphs based on the presence of different phyla in scenarios.

There were some taxa that were either found in very low abundance or exclusively in some of the scenarios. Three phyla, *Spirochaetes*, *Thermi*, and *Euryarchaeota*, were exclusively present in ScI and ScII (Figure 6). *Chitinophagaceae* family of *Bacteriodetes* was found higher in CSA based scenarios in general and specifically in maize-based scenarios, whereas the *Gaiellaceae* family was exceptionally higher in farmers’ practice (data not shown).

### Effect of Management Scenarios on Soil Chemical Properties and Available Nutrients

Soil properties like EC, pH, organic carbon, available macronutrients (N, P, and K) and micronutrient cations (Zn, Cu, Fe, and Mn) were analyzed for all the six scenarios (Table 3). Soil pH was found highest in ScIII and ScVI (7.70 ± 0.07 and 7.74 ± 0.06) and lowest in ScII (7.07 ± 0.04). SOC was highest in ScV (13.1 ± 0.02 g kg^–1^) followed by ScIV (12.5 ± 0.03 g kg^–1^) and ScVI (11.9 ± 0.01 g kg^–1^). SOC was 111% higher in CSA based scenarios (ScIII to ScVI) compared to farmers’ practice (ScI) and 31% higher than partial CA practice (ScII). Available nitrogen (N) was found highest (171 ± 1.00 kg ha^–1^) in ScIII and ScV, followed by ScVI (157 ± 1.53 kg ha^–1^) and ScIV (156 ± 3.21 kg ha^–1^). It was 38% and 14% higher in CSA based scenarios than ScI and ScII, respectively. Available phosphorus (P) was highest in ScV (31 ± 1.34 kg ha^–1^) and ScIV (30 ± 1.04 kg ha^–1^). It was 70% higher in CSA based scenarios than ScI and 18% higher than ScII. Available K was found highest (226 kg ha^–1^) with ScII and ScIV; it was 59% higher in CSA based scenarios over farmers’ practice (ScI). Among micronutrient cations, Zn was higher in ScIII and ScV, Mn was in ScIII, ScIV and ScV and Fe in ScII.

**TABLE 3:** Soil Chemical properties and available nutrients in soils after wheat harvesting.

**Scenario^a^**	**pH**	**EC (dS m^–1^)**	**SOC (g kg^–1^)**	**N (kg ha^–1^)**	**P (kg ha^–1^)**	**K (kg ha^–1^)**	**Zn (mg kg^–1^)**	**Cu (mg kg^–1^)**	**Fe (mg kg^–1^)**	**Mn (mg kg^–1^)**
ScI	7.41 ± 0.09b^b^	0.35 ± 0.02a	5.7 ± 0.01d	119.01 ± 0.58d	16.06 ± 1.04c	137.1 ± 1.15c	7.7 ± 0.15b	2.9 ± 0.12a	43.7 ± 1.81 b	53.4 ± 1.22c
ScII	7.07 ± 0.04c	0.34 ± 0.01a	9.2 ± 0.03c	144.12 ± 2.00c	23.1 ± 1.06b	225.5 ± 1.00a	8.8 ± 0.44ab	3.9 ± 0.15a	74.9 ± 1.46a	60.5 ± 0.83b
ScIII	7.70 ± 0.07a	0.32 ± 0.02a	10.7 ± 0.02b	171.07 ± 1.00a	24.92 ± 0.79b	217.8 ± 1.53b	9.7 ± 0.35a	2.8 ± 0.15a	23.1 ± 0.87c	66.8 ± 1.71a
ScIV	7.49 ± 0.02b	0.32 ± 0.03a	12.5 ± 0.03ab	155.91 ± 3.21b	29.7 ± 1.04a	225.5 ± 2.31a	3.7 ± 0.32c	2.7 ± 0.21a	20.6 ± 0.44c	72.4 ± 1.31a
ScV	7.49 ± 0.06b	0.28 ± 0.02a	13.1 ± 0.02a	171.32 ± 2.31a	30.52 ± 1.34a	210.1 ± 3.61b	10.1 ± 0.44a	2.6 ± 0.31a	21.5 ± 0.74c	68.6 ± 1.87a
ScVI	7.74 ± 0.06a	0.27 ± 0.03a	11.9 ± 0.01ab	157.25 ± 1.53b	24.20 ± 2.00b	217.8 ± 3.79b	3.6 ± 0.46c	2.3 ± 0.21a	15.2 ± 1.15c	48.8 ± 1.55c

**Where*; ScI, conventional rice-wheat system; ScII, partial CA based rice-wheat-mungbean system; ScIII, partial CSA based rice-wheat-mungbean system; ScIV, partial CSA based maize-wheat-mungbean system; ScV, full CSA based rice-wheat-mungbean system with sub surface drip irrigation; ScVI, full CSA based maize-wheat-mungbean system with sub surface drip irrigation. ^*a*^Refer Table 1 for scenario description. ^*b*^Means followed by the same letters within each column are not statistically different (*p* ≤ 0.05, Duncan’s multiple range test). Values are the average of three replicates (*n* = 3); means ± standard error SE. EC, electrical conductivity; SOC, soil organic carbon; N,P, K, available nitrogen, phosphorus and potassium; Zn, Cu, Fe, Mn, DTPA extractable zinc, copper, iron and manganese.*

### Relationships Between Bacterial Phyla and Soil Chemical Properties

**FIGURE 7:**
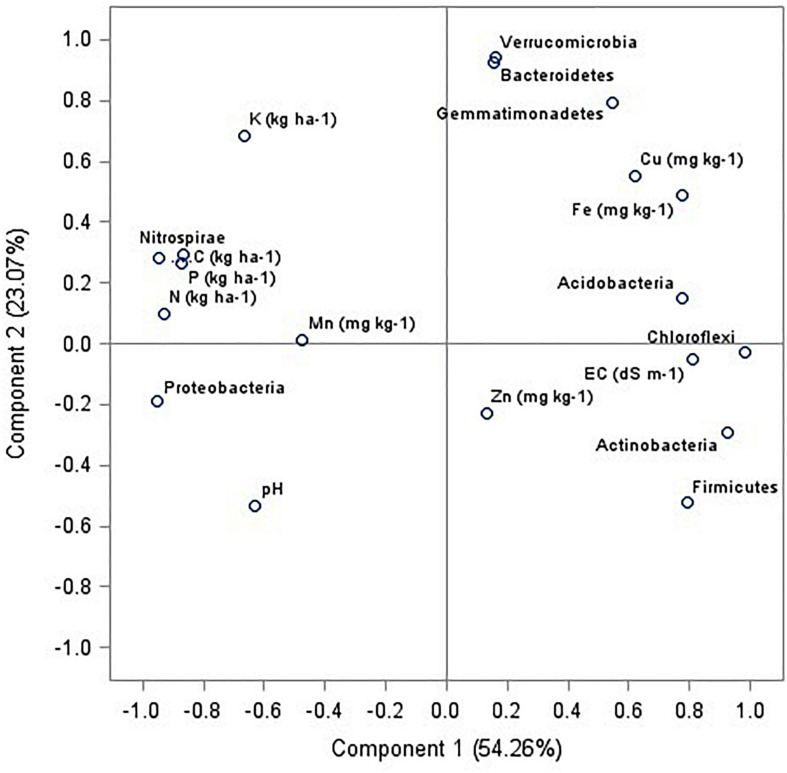
Principal component analysis among the soil properties and dominating bacterial phyla of six agricultural management systems.

The relationships between soil bacterial phyla (top 9) and soil chemical and available nutrients (pH, EC, C, N, P, K, Zn, Cu, Mg, Fe, and Mn) were examined using principal component analysis (PCA). Four principal components (PCs) with eigenvalues > 0.9 were extracted representing 96.7% of the total variance (Table 4). The PCA indicated that axis 1 (PC1) accounted for 54.3% and axis 2 (PC2) accounted for 23.1% of the total variance (Figure 7), whereas PC3 and PC4 showed 14.1 and 5.2% of the total variance (Table 5). In PC1, there were nine variables (*Proteobacteria*, *Actinobacteria*, *Chloroflexi*, *firmicutes*, *nitrospirae*, EC, SOC, N, K) with higher loadings (eigen vector > 0.8). To avoid the redundancy correlation study (Pearson’s correlation) was done among the nine variables (data not shown). The highest correlation sum was observed under C followed by N, K, and *Nitrospirae*. These four variables from PC1 were selected for the minimum dataset. In PC2, *Bacteroidetes*, *Verrucomicrobi*a, and *Gemmatimonadetes* were the important variables with higher loadings whereas in PC3, and PC4, Mn and Zn were selected. Therefore, among the nineteen variables nine variables namely SOC, N, K, Mn, Zn and *Nitrospirae*, *Bacteroidetes*, *Verrucomicrobia*, and *Gemmatimonadetes* were the most critical parameters as influenced by management practices.

**TABLE 4:** Principal components (PC) and component loadings extracted from different soil properties and microbial phyla; underlined component loadings were used to interpret the PC.

**PCs**	**PC1**	**PC2**	**PC3**	**PC4**
Eigen value	10.314	4.383	2.681	0.994
% Variance	54.284	23.069	14.085	5.230
Cumulative%	54.284	77.354	91.465	96.695
**Factor loading/eigen vector**				
*Proteobacteria*	**0.838**	–0.519	0.099	0.131
*Acidobacteria*	–0.629	0.374	–0.538	–0.405
*Actinobacteria*	−**0.964**	0.086	–0.039	0.181
*Bacteroidetes*	0.209	**0.902**	–0.132	–0.160
*Chloroflexi*	−**0.890**	0.343	–0.189	0.146
*Firmicutes*	−**0.971**	–0.192	0.031	–0.015
*Gemmatimonadetes*	–0.179	**0.920**	–0.269	–0.133
*Verrucomicrobia*	0.199	**0.906**	–0.159	–0.323
*Nitrospirae*	**0.815**	–0.063	0.337	–0.392
pH	0.424	–0.765	–0.286	–0.271
EC	−**0.838**	0.278	0.295	0.178
SOC	**0.927**	–0.094	0.295	–0.185
N	**0.912**	–0.228	0.238	0.198
P	0.793	–0.059	0.578	–0.098
K	**0.841**	0.397	0.259	–0.046
Zn	–0.097	–0.082	0.136	**0.978**
CU	–0.360	0.789	0.192	0.444
Fe	–0.497	0.775	–0.003	0.390
Mn	0.276	–0.113	**0.937**	0.165

*Where EC, electrical conductivity; SOC, soil organic carbon; N,P, K, available nitrogen, phosphorus and potassium; Zn, Cu, Fe, Mn, DTPA extractable zinc, copper, iron and manganese. Bold letter denotes variables with higher factor loading.*

**TABLE 5:** Relationships between bacterial phyla and soil properties in six scenarios of agriculture management.

	**Proteobacteria**	**Acidobacteria**	**Actinobacteria**	**Bacteroidetes**	**Chloroflexi**	**Firmicutes**	**Gemmatimonadetes**	**Verrucomicrobia**	**Nitrospirae**
pH	0.70	–0.26	–0.56	–0.43	–0.68	–0.23	–0.71	–0.51	0.34
EC (dS m^–1^)	–0.79	0.43	0.80	0.11	0.75	0.81	0.24	–0.05	–0.75
SOC (kg ha^–1^)	0.83	–0.71	−0.93**	0.07	−0.92**	−0.88*	–0.29	0.12	0.96**
N (kg ha^–1^)	0.94*	−0.85*	−0.90*	–0.03	−0.93**	–0.82	–0.49	–0.14	0.72
P (kg ha^–1^)	0.74	–0.81	–0.78	0.00	−0.82*	–0.76	–0.31	0.06	0.92**
K (kg ha^–1^)	0.53	–0.47	–0.84	0.59	–0.71	−0.85*	0.11	0.47	0.70
Zn (mg kg^–1^)	0.10	–0.45	0.27	–0.30	0.19	0.08	–0.21	–0.43	–0.39
Cu (mg kg^–1^)	–0.63	0.25	0.47	0.58	0.60	0.21	0.66	0.46	–0.49
Fe (mg kg^–1^)	–0.77	0.44	0.62	0.52	0.77	0.32	0.75	0.48	–0.60
Mn (mg kg^–1^)	0.41	–0.78	–0.30	–0.17	–0.45	–0.21	–0.44	–0.25	0.46

*Significance at the *p* < 0.01, **significance at the *p* < 0.001, ***significance at the *p* < 0.0001.

Correlation study (Pearson’s correlation) among all the nineteen variables was performed and the results showed that the main phyla were correlated (positively or negatively) with soil nutrients (Table 5). In particular, SOC was significantly positively correlated with *Nitrospirae* (*r* = 0.96, *p* < 0.05) whereas significantly negatively correlated with *Actinobacteria* (*r* = 0.93, *p* < 0.05), *Chloroflexi* (*r* = 0.92, *p* < 0.05), and *Firmicutes* (*r* = 0.88, *p* < 0.05). Others did not have any relationship with SOC. Available N was strongly positively correlated with the phylum *Proteobacteria* (*r* = 0.94, *p* < 0.05) and negatively correlated with *Acidobacteria* (*r* = 0.85, *p* < 0.05), *Actinobacteria* (*r* = 0.90, *p* < 0.05) and *Chloroflexi* (*r* = 0.93, *p* < 0.05). *Chloroflexi* and *Nitrospirae* were negatively and positively correlated with available P, respectively.

## Discussion

The rarefaction curve (Figure 1) shows that sequenced samples contain many common, readily detectable OTUs and plateau has not been reached because of the possibility that some OTUs may not have been predicted. Different agriculture practices influence soil bacterial diversity and community profiles (Navarro-Noya et al., 2013; Lupwayi et al., 2017; Choudhary et al., 2018d; Zhu et al., 2018). An increase in bacterial diversity with tillage is reported by some researchers (Lienhard et al., 2014; Degrune et al., 2016) and also confirmed in our previous study (Choudhary et al., 2018d), while there are also reports of increased bacterial diversity under zero tillage and residue retention (Ceja-Navarro et al., 2010; Constancias et al., 2014; Wang et al., 2019). Under tillage practices, due to soil disturbance and mixing of crop residues in soils, alteration in the distribution of nutrients can occur, resulting in more bacterial diversity under tillage based scenarios (ScI and ScII) than CSA based practices. Due to the lack of disturbance under no-tillage conditions in soils of CSA based practices for a long time (9 years), a type of equilibrium has been established between different bacterial taxa (Tyler, 2019) which leads to the lower diversity indices in CSA based scenarios (Table 2). Although soil samples were taken after the same crop (wheat) in all scenarios, the effect of preceding crops also create differences in bacterial diversity (Hilton et al., 2018). Higher diversity indices in maize-based CSA systems over rice-based can be attributed to the difference in their roots exudates and rhizodeposits (Sasse et al., 2018). Chemical composition of the cell wall of different crop residues (e.g., maize vs. rice) also can leave a pre-crop imprint on soil microbiome (Venter et al., 2016; Babin et al., 2019). Chemical energy and nutrients release during decomposition of crop residue varies with plant species (Jensen et al., 2005) and they also can influence soil microbial communities.

Agriculture management systems directly affect soil environments which in turn affect soil microbial community structures (Dong et al., 2017). In different agricultural management practices *Proteobacteria, Acidobacteria*, *Actinobacteria*, *Bacteroidetes*, and *Cholroflexi* are known to be among the dominating phyla (Degrune et al., 2016; Chen et al., 2018; Tyler, 2019; Wang et al., 2019). In our study, *Proteobacteria* was the dominating phylum, but its relative abundance was higher in CSA based scenarios compared to those of partial CA and farmers’ practice (Figure 3). *Proteobacteria* are copiotrophic in nature, which thrive better under conditions of high nutrient availability (Ho et al., 2017). *Proteobacteria* are known to be highly abundant in soils having minimum tillage, whereas *Acidobacteria*, *Actinobacteria*, and *Cholroflexi* to be in conventional tillage (Legrand et al., 2018). Relatively higher abundance of *Acidobacteria* and *Actinobacteria* were associated with tillage based scenarios (ScI and ScII) which were reported to have oligotrophic lifestyle and thrive better under lower nutrient availability conditions (Fierer et al., 2012; Koyama et al., 2014; Ho et al., 2017). As stated in a previous study (Choudhary et al., 2018d), the ratio of *Proteobacteria* and *Acidobacteria* (P/A) also provides insight into the soil nutrient status (Smit et al., 2001); a high P/A ratio would be indicative of a copiotrophic and a low one of oligotrophic nature of soils. In ScI and ScII the ratio of relative abundance of *Proteobacteria* and *Acidobacteria* was found lower than that of CA-based scenarios. In surface soil layer, SOC and macronutrients (N, P, and K) were found to be significantly higher in CA-based management practices than tillage based practices, which was also confirmed in the study of Patra et al. (2019). Moreover higher enzyme activities under CA/CSA-based scenarios than conventional practices (Jat et al., 2019a, 2020) facilitates the mineralization of nutrients that become available to microbes and favors copiotrophs more in CSA based practices. Under maize-based scenarios quality and size of maize residues may play a role in the lower relative abundance of *Proteobacteria* than in rice-based CSA scenarios as the type of residue has influence on microbial diversity (Nelson and Mele, 2006). The difference between crop types (maize/rice) also has a major role in determining the type of bacterial communities in soils. The study of chloroplast genome revealed a closer relationship between rice and wheat than maize (Matsuoka et al., 2002) and this evolutionary history of plants can be a significant factor in the shaping of associated bacterial community composition (Bouffaud et al., 2014). Every plant species host distinct bacterial community in which rhizodeposition also play a major role (Bulgarelli et al., 2013).

The phylum *Chloroflexi* was recorded higher in ScI and ScII; members of this phylum contain varied phenotypes with wide-ranging metabolic lifestyles, aerobic thermophiles, photoautotrophs, and anaerobic halorespires (Hug et al., 2013). Members of *Chloroflexi* are also oligotrophic and reported to have a role in cellulose degradation and C-cycling (Cole et al., 2013; Pepe-Ranney et al., 2016). Under conventional tillage practices, soil disturbance occurs and also in the absence of residue cover more chances of drying of soil occurs in farmers’ practice as compared to CA practices. Firmicutes are known to produce endospores (Mandic-Mulec and Prosser, 2011) which make them able to survive in disturbed conditions caused by tillage and uncovered soil surface. Hence, their relative abundance is higher under conventional tillage than zero tillage practices (Lienhard et al., 2014).

Higher abundance of *Alphaproteobacteria* in ScIII and ScIV can be attributed to long-term CSA practices (9 years) followed in these scenarios which created a congenial environment that favored *Alphaproteobacteria*. Whereas a higher abundance of *Gammaproteobacteria* in CA-based scenarios may be due to the ZT practices followed in these scenarios (Essel et al., 2018). *Pseudomonadales* were favored in CSA practices which consist of bacteria like *Azotobacter* and *Pseudomonas* having plant growth-promoting activities (Nehra and Choudhary, 2015). Some of the *Pseudomonas* species are also known as plant pathogenic (Höfte and De Vos, 2007). CSA practices also favored *Rhizobiales* which is a well-known order consisting of the members of atmospheric nitrogen-fixers and are symbiotic with plant roots. Quality, quantity, and management of residue are differed between scenarios, which may be important factors affecting the structure of bacterial communities (Jiménez-Bueno et al., 2016). Crop residues are considered as a crucial ecological niche of different microbial communities. The order *Rhizobiales* and *Sphingomonadales* were found prevalent in the wheat-oilseed rape cropping system irrespective of the type of residues (Kerdraon et al., 2019). Members of the *Chitinophagaceae* family are known for the decomposition of complex organic materials and reported to be more abundant in no-till than conventional-till (Kraut-Cohen et al., 2020). Members of the *Gaiellaceae* family favor elevated levels of oxygen (Albuquerque and da Costa, 2014) and are exceptionally higher where regular tillage is practiced. During hot summer, puddling operation (churning of soil in the presence of water) exposes the soil to high sunlight and heat in the IGP. *Deinococcus–Thermus*, having thermophilic properties and being resistant to radiations (Griffiths and Gupta, 2007), were exclusively present in scenarios where tillage practice followed (ScI and ScII). *Methanomicrobia* and *Methanobacteria* were the two classes of *Euryarchaeota* (Archaeabacteria) present in tillage based systems as in these scenarios rice crop was established by transplanting (Table 1) and water stagnates for a longer time which creates anaerobic condition and favors these two classes.

Beta diversity showed (Figure 2) that ScIII, IV, V, and VI are closely related to each other and distantly related to ScI and II, which is the reflection of management followed in these scenarios. Varying/inconsistent results were obtained at lower classification levels which may be because of many different factors associated with different crop rotations, tillage, and water and residue management directly or indirectly influencing microbial community structure (Ramirez-Villanueva et al., 2015; Venter et al., 2016; Wang et al., 2016; Babin et al., 2019). In addition to these factors root exudates associated with various crops also have different impacts on soil microbial communities (Sasse et al., 2018). Conservation agriculture practices improve soil physicochemical properties and nutrient availability (Jat et al., 2018) which could be also a reason behind different types of distribution of bacterial groups among scenarios.

A significant variation in soil pH was observed among the scenarios. The lowest pH was observed in ScII, which might be due to the well mixing of crop residues into the soil during puddling operation before rice transplanting, which releases the organic acids (Ghimire et al., 2017), facilitating a pH drop in soil. SOC was higher in CSA based scenarios which can be attributed to the higher quantity of carbon input through crop residue retentions under CSA practices, since the initiation of the experiment (Jat et al., 2018, 2019a). Less soil disturbance conditions under zero tillage (ZT) resulted in slow decomposition of crop residue which is also a factor responsible for high C and N under CSA practices (Dikgwatlhe et al., 2014). The availability of macronutrients (N, P, and K) is significantly influenced by agriculture practices (Zhang et al., 2018; Jat et al., 2018; Sithole and Magwaza, 2019); in our study, higher N, P and K were observed under CSA practices. Higher amounts of residues under CSA practices serve as a source of plant nutrients (Bhattacharjya et al., 2019) moreover mungbean integration in these cropping systems also increases the availability of macro and micronutrients (Jat et al., 2018). The DTPA extractable Fe content was much higher than the critical Fe concentration in soil (4.5 mg/kg) under CSA practices (ScIII-VI) but it was lower than tillage based scenarios (ScI-II). It might be due to the oxidation of Fe under the aerobic condition which facilitates the conversion of ferrous (Fe^2+^) to ferric (Fe^3+^) and gets precipitated and becomes unavailable to plants (Lovley, 1995; Sparrow and Uren, 2014). But in ScI and ScII, rice was grown in the submerged puddled condition which keeps the iron in reduced soluble form in the soil (Pezeshki and DeLaune, 2012) which might enhance higher recovery of DTPA-Fe from soil. Conservation agriculture-based management practices improve soil physical, chemical, and biological properties of soil and in turn soil quality (Raiesi and Kabiri, 2016; Choudhary et al., 2018b). Improved soil quality leads to an increase in the availability of soil nutrients (Wang et al., 2019). Soil properties such as SOC, macronutrients (N and K), and micronutrients (Zn and Mn) were the important variables that significantly influenced microbial dominance (such as *Nitrospirae*, *Bacteroidetes*, *Verrucomicrobia*, and *Gemmatimonadetes*) as revealed by PCA. Higher carbon inputs through crop residues (and roots, rhizodeposition etc.) coupled with ZT enhanced SOC and N content which favor the biological activities and microbial colony counts (Choudhary et al., 2018a, b; Jat et al., 2019a). Crop residues provide K, Zn, and Mn and serve as a food source to microorganisms, allowing the flourishing of the populations of certain phyla. Choudhary et al. (2018a) reported microbial biomass carbon as one of the key indicators in the soil, while studying soil quality index under CA, which is a sensitive indicator of change in soil organic matter levels (Gregorich et al., 1994). Land and agriculture management practices influenced all the soil chemical properties (Parisi et al., 2005; Van Leeuwen et al., 2015) which directly or indirectly have strong bearings on soil microbial phyla dominance.

Since *Acidobacteria* and *Actinobacteria* are oligotrophic in nature, they showed a negative correlation with SOC and available N. *Proteobacteria*, which are of copiotrophic nature, showed a positive correlation with available N. A study on bacterial diversity in four scenarios out of six scenarios has been done previously after 6 years of continuous experimentation (Choudhary et al., 2018c, d). Although the result of diversity indices of this study was similar to our previous study (Choudhary et al., 2018d), the differences were observed at different classification levels (phylum, class, order). In the previous study, *Acidobacteria* was the most dominant phylum in the CA-based scenario followed by *Proteobacteria*, and in other scenarios, *Proteobacteria* was dominating followed by *Acidobacteria*. At the class level, *Alphaproteobacteria* was the most abundant class followed by *Acidobacteria- 6* in all scenarios except in ScIV. The most abundant class in ScIV was *DA052* followed by *Alphaproteobacteria*. There are many reasons behind the different patterns of bacterial diversity in the same soils under similar conditions (Quince et al., 2017). It may be due to the difference between sampling dates and sample size. In the previous study, sampling was done in mid-May and in the present study, it was done in mid-April which can cause a difference in soil moisture, temperature, and other factors. The previous study was done from composite soil samples which may have masked differences that varied between management systems (Tyler, 2019). The contribution of the specific phylum or group in the agroecosystem is very important, but still, the role of microbial diversity in functioning and sustainability of the agroecosystem is poorly understood (van der Heijden and Wagg, 2013). Despite the occurrence of many genera or species in different management practices, which are important contributors in a variety of ecosystems, their potential ecological roles in the soil remain unknown. In the coming years with the advancement of techniques and wider studies, in-depth roles of these will be unraveled.

## Conclusion

Conservation agriculture coupled with precision water and nutrient management in intensive cereal based cropping systems serves as fully validated Climate Smart Agriculture (CSA). The crucial role of soil bacterial composition & diversity and their interactions with available soil nutrients further provides insights for building resilience against climatic risks. A higher relative abundance of copiotrophs (*Proteobacteria*) was found in CSA based agri-food systems while oligotrophs (*Acidobacteria* and *Actinobacteria*) were associated with conventional tillage (CT) based rice-wheat system. The bacterial diversity indices were directly correlated with the intensity of tillage & system management practices with higher diversity under CT and lower with CSA scenarios. In surface soil layers, soil organic carbon (SOC) and major nutrients (N, P, and K) were significantly higher in CSA practices than business-as-usual (CT based rice-wheat). Soil organic carbon and available- P were found positively correlated with the *Nitrospirae* whereas available- N was with the *Proteobacteria* indicating a significant role of CSA based management practices in nutrient cycling and plant availability.

## Data Availability Statement

The datasets generated for this study can be found in the NCBI Sequence Read Archive (SRA) under the BioProject: PRJNA563825.

## Author Contributions

HJ, PS, and MJ conceptualized and designed the experiment. MJ and PS did the funding acquisition. HJ and PS did the management of the experiment. HJ, AD, and MC conducted the research. MC, AD, and BR analyzed the data. MC wrote the manuscript. HJ, MJ, and AD provided critical comments on the manuscript and participated in the revision. All authors read and approved the final manuscript.

## Conflict of Interest

BR was employed by Celixa.

The remaining authors declare that the research was conducted in the absence of any commercial or financial relationships that could be construed as a potential conflict of interest.

## Footnotes

1 http://www.bioinformatics.babraham.ac.uk/projects/fastqc/
